# Persistent SARS-CoV-2 Nucleocapsid Protein Presence in the Intestinal Epithelium of a Pediatric Patient 3 Months After Acute Infection

**DOI:** 10.1097/PG9.0000000000000152

**Published:** 2021-12-03

**Authors:** Dalia Arostegui, Kenny Castro, Steven Schwarz, Katherine Vaidy, Simon Rabinowitz, Thomas Wallach

**Affiliations:** From the SUNY Downstate Health Sciences University, Brooklyn, NY.

**Keywords:** SARS-CoV-2, long COVID, COVID

## Abstract

In addition to the severe impact of acute respiratory disease during the SARS-CoV-2 pandemic, the issue of “Long COVID” illness has impacted large numbers of patients following the initial infection. Wide ranges of Long Covid incidence have been reported, ranging from 30 to 87%. Long COVID has a variety of clinical manifestations, including gastrointestinal symptoms. Here, we report a case of persistent abdominal pain, 3 months following a SARS-CoV-2 diagnosis, associated with chronic colonic inflammation and the presence of mucosal SARS-CoV-2 virions.

## INTRODUCTION

The coronavirus SARS-CoV-2 is a novel single-stranded RNA beta-coronavirus identified in Wuhan, China, in December 2019 (1). The rapid spread of SARS-CoV-2 disease has resulted in >4 million deaths worldwide (2). In addition to respiratory compromise, SARS-COV-2 may induce gastrointestinal symptoms such as diarrhea and vomiting (3). Beyond the acute viral illness, so called “long COVID” (LC) disease has been described, with symptoms persisting well beyond the initial period of infection (4). Variations of LC may impact over 80% of patients (5). The pathophysiology behind this latter phenomenon is unclear, although hypothesized to be similar to postviral syndromes driven by agents such as Epstein-Barr and Herpes Simplex viruses (6–8). Others suggest LC may represent the effects of persistent viral presence after acute infection (9,10).

The report herein describes the case of an 11-year-old female with chronic abdominal pain following a PCR-positive COVID infection. Because of continued symptoms, upper gastrointestinal tract endoscopy and colonoscopy were performed 3 months following her initial SARS-CoV-2 diagnosis. Histopathology of biopsied colonic tissue demonstrated a dense lymphocytic infiltrate, and immunohistochemical staining identified SARS-CoV-2.

## CASE PRESENTATION

An 11-year-old female with a history of SARS-CoV-2 infection, confirmed by a positive polymerase chain reaction (PCR), presented to the pediatric gastroenterology clinic at the Children’s Hospital at Downstate, SUNY-Downstate Health Sciences University, with intermittent periumbilical, right upper and lower quadrant abdominal pain worst after eating. These symptoms began during an acute SARS-CoV-2 infection 3 months earlier. She described the pain as cramping or burning, that attenuates with stooling or eating, and ranging in intensity from 5 to 7/10. Review of symptoms noted associated nausea but denied emesis, weight loss, fever, diarrhea, hematochezia, joint pains, or skin changes.

**FIGURE 1.**
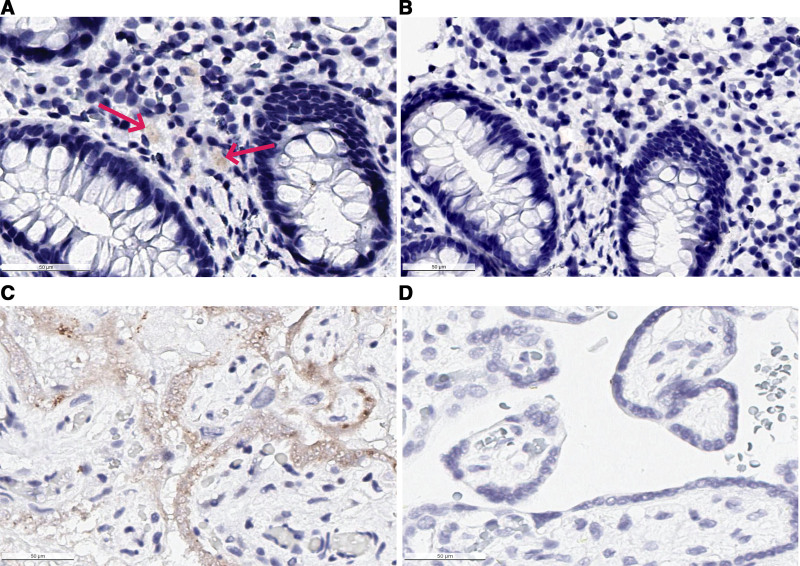
SARS-CoV-2 positive staining in colonic mucosa. A) Cecal biopsy after completion of hematoxylin and eosin staining as well as immunohistochemical staining with rabbit monoclonal SARS-CoV-2 nucleocapsid antibody. Brown staining indicates SARS-CoV-2 nucleocapsid presence. B) Negative control slide from the same cecal tissue block, prepared identically but without addition of primary SARS-CoV-2 nucleocapsid antibody demonstrating absence of staining. C) Positive control for SARS-CoV-2 nucleocapsid antibody stain in known SARS-CoV-2 positive placental tissue. D) Negative Control in known SARS-CoV-2 negative placental tissue.

Physical examination noted periumbilical and epigastric tenderness but no other notable findings. The patient was initially given a proton pump inhibitor (PPI) therapeutic trial. Laboratory workup included a complete blood count, comprehensive metabolic panel, vitamin D level, erythrocyte sedimentation rate, C-reactive protein, tissue transglutaminase antibody IgA, total IgA level, fecal *H. pylori* antigen and calprotectin. All studies were within normal limits, with the exception of vitamin D deficiency (14 ng/mL; normal >30) and an elevated fecal calprotectin (358 µg/g; normal <100). After 3 weeks of therapy, her abdominal pain was unresolved. Due to continued symptoms and elevation in fecal calprotectin, an esophagogastroduodenoscopy (EGD) and colonoscopy were performed. The EGD was endoscopically and histologically normal, while the colonoscopy demonstrated friability throughout the colon on visual examination. All upper gastrointestinal biopsies were unremarkable. However, colonic mucosal biopsies demonstrated a widespread lymphocytic infiltrate without other stigmata of inflammation or glandular distortion. Magnetic resonance enterography was obtained and was determined to be normal. Given her history of SARS-CoV-2, prolonged symptoms and colonic histological changes consistent with an ongoing viral diathesis, immunohistochemical staining for SARS-CoV-2 in the colon was performed as previously described, using a rabbit monoclonal SARS-CoV-2 nucleocapsid antibody (GTX635686, 1:10,000) (11). SARS-CoV-2 virions were identified in the cecum (Fig. 1). This finding demonstrates SARS-CoV-2 nucleocapsid proteins in the intestinal lamina propria 3 months after infection, suggesting a role for persistent viral infection in the pathogenesis of gastrointestinal LC.

## DISCUSSION

The present case demonstrates continued presence of SARS-COV-2 virions in the colon 3 months after acute infection, potentially linked to symptoms of chronic abdominal pain and the laboratory finding of an elevated fecal calprotectin. The presence of a dense lymphocytic infiltrate in colonic biopsies may therefore represent a response to persistent SARS-COV-2 infection, and strongly suggest ongoing viral colonization is involved in the etiopathogenesis of gastrointestinal-predominant LC.

Infection with SARS-COV-2 has been associated with effects both on the innate and on the adaptive immune systems (12), Virally induced release of IL-2, IL-7, TNF-alpha, granulocyte colony-stimulating factor, and interferon gamma (13), are well known to mediate a GI inflammatory response and its attendant symptoms (14). In our patient, GI inflammation was evidenced both by an elevated fecal calprotectin and by the histologic finding of a significant lymphocytic reaction, consistent with an active viral process.

SARS-CoV-2 enters host cells primarily via two receptors, the metallopeptidase angiotensin converting enzyme 2 receptor (ACE2), and a protein coding gene called transmembrane serine protease 2 (TMPRSS2) (15). After viral binding to ACE2 receptors, TMPRSS2 allows for cleavage of the spiked glycoprotein on the viral envelope and will therefore allow penetration of the virus into host cells (16). ACE2 receptors are present in a range of human cell types vulnerable to viral infection, including not only the lung epithelium, but also small intestinal and colonic enterocytes, neuronal and glial cells (17–19). SARS-CoV-2 has been shown to replicate in an *in vitro* model of the human intestinal tract using C2BBe1 intestinal cells, which are known to express high levels of TMPRSS2 (20). In aggregate, these findings suggest the potential capacity of SARS-CoV-2 to infect intestinal epithelial cells long-term, as one possible source of persistent SARS-CoV-2 presence (20).

As previously discussed, ACE2 receptors also allow for SARS-CoV-2 infection of neurons. As SARS-CoV-2 has shown to be able to both infect neuronal tissue (21) and directly impact ENS function (22,23), we speculate prolonged gastrointestinal symptoms following SARS-CoV-2 infection are driven by viral persistence in ENS cells. Multiple viruses have demonstrated the capacity to create latent or chronic infection in ENS cells, leading to intestinal inflammation and dysfunction (24,25). As the ENS is directly connected to the intestinal epithelium via enterochromaffin cells (26), sparse reinfection of the intestinal epithelium and intestinal motor and sensory dysfunction could drive this patient’s symptoms and mild inflammatory picture.

The case described in this report strongly suggests that prolonged gastrointestinal symptoms following SARS-CoV-2 diagnosis, with evidence of chronic inflammation 3 months post-diagnosis, are the direct consequence of SARS-CoV-2. Such long-term, symptomatic SARS-CoV-2 infection may demonstrate the need for LC therapeutic interventions, including vaccination (27), monoclonal antibody therapy (28,29), or corticosteroid therapy (30). Certainly, further studies are needed to evaluate the impact and frequency of persistent gastrointestinal SARS-CoV-2 infection, and to correlate symptoms with the severity of disease.
